# Ketogenic Diet in Cancer Prevention and Therapy: Molecular Targets and Therapeutic Opportunities

**DOI:** 10.3390/cimb43020042

**Published:** 2021-07-03

**Authors:** Wamidh H. Talib, Asma Ismail Mahmod, Ayah Kamal, Hasan M. Rashid, Aya M. D. Alashqar, Samar Khater, Duaa Jamal, Mostafa Waly

**Affiliations:** 1 Department of Clinical Pharmacy and Therapeutics, Applied Science Private University, Amman 11931, Jordan; asmamahmod1212@gmail.com (A.I.M.); ayakamal257@gmail.com (A.K.); Hasanalzubaidy08@gmail.com (H.M.R.); ayaalashqar1995@gmail.com (A.M.D.A.); samar_ktr@asu.edu.jo (S.K.); jdouaa94@gmail.com (D.J.); 2 Department of Food Science and Nutrition, College of Agricultural and Marine Sciences, Sultan Qaboos University, Al-Khoud 34-123, Oman; mostafa@squ.edu.om

**Keywords:** alternative cancer therapies, anticancer diet, targeting cancer metabolism, calories restriction

## Abstract

Although cancer is still one of the most significant global challenges facing public health, the world still lacks complementary approaches that would significantly enhance the efficacy of standard anticancer therapies. One of the essential strategies during cancer treatment is following a healthy diet program. The ketogenic diet (KD) has recently emerged as a metabolic therapy in cancer treatment, targeting cancer cell metabolism rather than a conventional dietary approach. The ketogenic diet (KD), a high-fat and very-low-carbohydrate with adequate amounts of protein, has shown antitumor effects by reducing energy supplies to cells. This low energy supply inhibits tumor growth, explaining the ketogenic diet’s therapeutic mechanisms in cancer treatment. This review highlights the crucial mechanisms that explain the ketogenic diet’s potential antitumor effects, which probably produces an unfavorable metabolic environment for cancer cells and can be used as a promising adjuvant in cancer therapy. Studies discussed in this review provide a solid background for researchers and physicians to design new combination therapies based on KD and conventional therapies.

## 1. Introduction

Cancer, “the sickness of the century”, is one of this era’s leading causes of mortality worldwide, which is becoming more threatening day by day due to the increasing number of cancer cases and the ability of this disease to resist the existent therapeutic and pharmacological approaches. In the year 2020 and in the United States alone, 1,806,590 new cancer cases were discovered according to the American Cancer Society, with 606,520 cancer deaths [1]. With approximately 89,500 new cancer cases per year and 9270 deaths in adolescents and young adults [2]. The conventional interventions such as surgery, chemotherapy, hormonal therapy, radiation therapy, monoclonal antibodies, and tyrosine kinase inhibitors were able to eliminate some types of cancers, induce cancer regression and inhibit the growth of some others [3]. Nevertheless, each one of these interventions has its own limitations that can be an obstacle to both healthcare providers and patients to reach the desirable objectives; for example, the advanced stage of cancer and metastasis level renders the surgical procedure unreasonable and not effective. In addition, both chemotherapy and radiation therapy have cancer induction effects that may lead to secondary tumors and various toxicity issues inducing normal-tissue complications; these factors and others put pressure on the medical body to find new, safe, and cost-effective cancer therapy agents [3].

The metabolic differences between normal cells and cancerous cells is not a new subject, especially after the discovery of the Warburg effect by Otto Warburg in 1920s [4,5,6], and the formulation of his hypothesis in 1956. Based on his hypothesis, cancer cells have irreversible damage in cell respiration and dysfunction in the mitochondria, making them dependent on fermentation to obtain ATP [7,8,9,10]. Further studies suggested that this process is more important for the production of substantial building blocks for cancer, which means it is associated with cell proliferation and cancer growth [11,12].

Consequently, in the last few years, there has been a strong direction by researchers to find or develop a diet-based strategy as a new complementary therapy that affects cancer cells’ metabolic pathways. Intermittent fasting, prolonged fasting, ketogenic diet (KD), fasting-mimicking diet and alternative caloric restrictions are dietary approaches that are being studied with many clinical and animal trials available to prove their efficacy and ability to, at least, be used as prophylactic or adjuvant strategies in cancer treatment [13,14,15,16,17,18,19].

A ketogenic diet (KD) causes a metabolic shift from glycolysis into mitochondrial metabolism, the differential stress resistance phenomenon with high tumor control ability and lower normal-tissue complications [17], which makes the ketogenic diet an interesting dietary approach for cancer patients under supervision and follow up of a healthcare provider. The ketogenic diet content is distinguished by high fat, moderate to low protein and very low carbohydrate intake. The macronutrient distribution of KD is about 90% fat, 2% carbohydrate, and 8% protein. This is achieved by following the standard fat to carbohydrate and protein ratio of 4:1 and 3:1, respectively [20]. Another recent study has suggested that the clinical use of KD is composed of at least 80% fat with a KD ratio of 2:1 to 3:1 [21]. The low intake of glucose accompanied by high fat and adequate protein content causes a reduction in IGF1 and an increase in ketosis or ketone body production in human clinical studies [18]. Moreover, multiple studies have also supported that carbohydrate restriction has a protective effect against cancer in animal models [22,23,24,25]. The use of a ketogenic diet was reported to show improvement in a patient condition in one of two girls with advanced astrocytoma, and this observation may be explained on the basis that brain tumors are incapable of using ketones as an energy source in comparison with healthy brain tissue [26]. However, results from other clinical studies indicated that sufficient therapeutic activity was not achieved when a ketogenic diet was used as the only treatment in patients with cancer. These results suggest that to achieve the potential benefits of such diets, they should be used in combination with other treatment strategies, including chemotherapy, radiotherapy, antiangiogenic treatments, PI3K inhibitors, and fasting-mimicking diet [22,27].

In this review article, we will describe the history of the ketogenic diet, details concerning cancer metabolism and the Warburg effect, the mechanism of action for the ketogenic diet as a cancer therapy and the clinical and experimental studies regarding the use of KD as an anti-cancer therapy.

## 2. The History of Ketogenic Diet

In the past, based on the Hippocratic collection, the only therapeutic measure against epilepsy was fasting [28], and in the 5th century BC, complete fasting was prescribed, and was known to be effective. The ketogenic diet was established early in the 20th century as an approach to mimic the effects of fasting; Marie and Guelpa published a study on the effect of fasting in epileptic patients [29], which reported less severe seizures observed during such a treatment, without further detail. In addition, contemporary reports regarding fasting were also recorded early in the 20th century in the USA, with a report on a patient of Dr. Hugh W. Conklin being the first, and another belonging to Bernarr Macfadden [30,31].

Macfadden was a physical fitness cultist [32] who established his first magazine, Physical Culture, in 1899, in which he directed the readers to maintain their health and to deal with sickness through diet and exercise; the magazine was widespread by the end of World War I. Macfadden emphasized fasting and his rationale was that much of the body’s energy is used in food digestion; as a result, if there is no food to digest, more energy could be used to recover health. Macfadden, who was recognized nationally back then, assumed that fasting for three days up to 3 weeks could be a cure for almost any disease, including liver and kidney disease, diabetes, bladder disease, asthma, epilepsy, prostate disease, paralysis, and impotence [32].

Macfadden’s assistant, Dr. Conklin, adopted his method of [33] using fasting to treat epilepsy in his practice, with results that drew other pioneers into epilepsy studies, such as H. Rawle Geyelin, who was an endocrinologist at the New York Presbyterian Hospital. Geyelin first reported his exposure to fasting as a treatment for epilepsy at the American Medical Association (AMA) conference in 1921 [34]. His report was based on an observation of a young cousin who had epilepsy for four years [35] and in spite of multiple recommended treatments, the patient failed to respond and his seizures were not controlled. Consequently, Dr. Conklin subjected the child to four periods of fasting over several months and observed the cessation of seizures after the second day of fasting; no seizures were observed over two years of follow-up. Dr. Geyelin began using the same fasting strategy after observing two other patients who had apparently been cured of epilepsy by Dr. Conklin, but initially used different fasting periods and finally decided to use a 20-day fasting period. Geyelin suggested that fasting can almost always be used as a method to clear a clouded mentality. This observation has received strength in recent years, after reports of an association between developmental and behavioral improvements and the ketogenic diet. Initially, Dr. Geyelin, in a preliminary report, documented the efficacy of fasting in patients and closed his presentation as further studies were needed [36].

News of Conklin’s successful therapy had spread to others in conventional neurology practices even before he published his results [33,37]. Moreover, Conklin’s fasting therapy was mentioned by Dr. Penfield and his colleague Dr. Erickson in their textbook on epilepsy published in 1941 [38]. Additionally, Lennox acknowledged that by 1928, Conklin had the most experience in using fasting for treating epilepsy patients, which lead others to adopt Conklin and Macfadden’s views on the origins and treatment of epilepsy [33].

Earlier in 1919, Dr. John Howland, professor of pediatrics at Johns Hopkins, used a grant from Charles Howland to determine the first laboratory to establish whether there was a scientific basis for the positive results of starvation treatment in epilepsy [39,40], and by 1937, it was used to support research on the KD [41]. Moreover, the first two physicians who confirmed and explained the effects of fasting on epilepsy were reported by Lennox. The first was Dr. James Gamble, who was recruited by Howland; in 1915, he reported a study on the acid–base balance of two children on a fasting program [42]. Furthermore, Gamble’s report established a model for clinical research and produced the fundamentals for pediatric electrolyte physiology and nephrology fields. In addition, he also reported an increase of calcium elimination in patients on the ketogenic diet, which necessitated calcium supplements [37]. H. Rawle Geyelin was the other doctor who was also recruited by Howland and presented his findings on fasting as a treatment of epilepsy at the 1921 AMA conference. Unfortunately, his data were never published, but he informed Lennox that in the long term, no seizures occurred in 19% of the treated children, while this was observed only in 0.5% for the treated adults [33,34]. Dr. Stanley Cobb, an associate professor of neuropathology at Harvard Medical School with the assistance from a colleague, W. G. Lennox, studied a selected group of five patients during a fasting period of two weeks [37], and throughout the study, chemical assays were performed on the blood and urine of both the subjects and controls. All the analyses showed an increase in blood acidosis and serum uric acid that was usually evident after two or three days of fasting and was accompanied by a decrease in seizures. These reports on the efficacy of fasting triggered a surge of clinical and research activity with a rise in theories that tried to offer an explanation for the therapeutic efficacy of starvation. Dehydration [42,43], ketosis [37,44,45,46], and acidosis [33,47,48] were all put forward as mechanisms that may explain the efficacy of fasting. Moreover, many investigators of that era used metabolic balance in an effort to understand the interrelationships of fat, protein, and carbohydrate metabolism to the disturbed glucose utilization and ketoacidosis that occurs in diabetes.

Similarly, in 1921, at the same time of the Cobb and Lennox study, in a review article about diet adjustments and diabetes, Woodyatt reported that starvation in normal subjects leads to the production of acetone, acetic acid, and beta hydroxybutyric acid, as well as being on a diet consisting of low carbohydrate content with a high fat content and that ketoacidosis appeared to be the immediate result of the oxidation of certain fatty acids in the absence of a sufficient proportion of ‘oxidizing’ glucose [49]. Simultaneously and possibly grounded on the work summarized by Woodyatt, Dr. Wilder at the Mayo Clinic suggested that to obtain the benefits of fasting, ketone bodies and thus ketonemia can be produced from fat and protein when there is variance between the amount of fatty acids and the amount of sugar burning in the tissues. The possibility to stimulate ketogenesis by consuming diets rich in fat and low in carbohydrate was well known. Therefore, it was proposed to test the effects of such ketogenic diets on epilepsy [46]. Moreover, Wilder also suggested that a ketogenic diet could be maintained for a much longer period than fasting with same effectiveness and eventually he was the first to formulate the ketogenic diet term. Later, Peterman with pediatricians eagerly acted on Wilder’s suggestion [50,51], and in 1924, Peterman reported the calculations and effectiveness of the KD from the Mayo Clinic [19,26,51]. The ketogenic diet being used today is identical to Peterman’s KD, which is composed of 1.0 g of protein per kilogram of body weight for children, 10 to 15 g of carbohydrate per day, and fat as the remainder of the calories. Peterman also reported his observation that the ketogenic diet leads to improvements in behavior and cognitive effects.

The first prospective study, “the effects of the KD on development and behavior was completed by M. B. Pulsifer and colleagues” in 2001 [36], revealed statistically significant behavioral improvements in social functioning and attention in children with intractable seizures, thus confirming the earlier observations of Peterman. Later reports from Talbot et al. in 1926 [52,53,54] and from McQuarrie and Keith in 1927 [55] followed those preliminary reports. Talbot reported the following: “In 1921, the Massachusetts General Hospital children’s medical service began a study on the treatment of idiopathic epilepsy. The first method used was the fasting recommended by Conklin” [53]. Consequently, the ketogenic diet that was carried out by the Mayo Clinic, which was described by Dr. Peterman, was adopted in 1924 by MGH. Moreover, Talbot, by the year 1928, had already used different compositions of ketogenic diets in his practice and reported that in order the achieve the best therapeutic outcomes in epilepsy, the ratio of 4:1 must be approached [37], which is acknowledged as the most common composition for the ketogenic diet in modern time. Moreover, variation in the level of ketosis that prevents seizures among individuals was recognized by McQuarrie and Keith which made it necessary to adjust the diet for individuals in order to reach the optimal ketosis.

The ketogenic diet was extensively utilized in the 1920s and throughout the 1930s, up until the discovery of the drug diphenylhydantoin by Merritt and Putnam in 1938, which lead to the withdrawal of researchers’ interest from the ketogenic diet mechanism of action and effectiveness to that of new antiepileptic drugs (AEDs). This marked the beginning of a new age of medical therapy for epilepsy, and the ketogenic diet was given up since medications were easier to administer and new chemicals were always at hand. Furthermore, in a pediatric neurology manuscript in 1937, Ford reported that the ketogenic diet was difficult to use, inflexible, and expensive [56], and as more AEDs became available, use of the ketogenic diet was phased out. In addition, sodium valproate was introduced and this branched-chain fatty acid was believed to be effective in the treatment of children with seizures of Lennox–Gas taut syndrome who were previously placed on the diet; thus, the diet could no longer be defended [57]. These findings resulted in a decline in the number of dieticians training for the initiation and maintenance of the diet. Although the ketogenic diet use greatly declined over the years, an NBC-TV Dateline program aired on October 1994 reinvigorated attention to the treatment [30,31,58,59] and was based on the true story of a two-year-old, Charlie, who suffered from generalized tonic, myoclonic, and tonic–clonic seizures. Charlie’s father, while researching treatments for epilepsy on his own, found a reference that linked the ketogenic diet to Johns Hopkins [60], to which Charlie was transported and was initiated on the diet, after which he became seizure-free and also displayed developmental progress. Later, in 1995, Wheless concluded that the KD works well in comparison with other new epilepsy treatments in children and should therefore be accessible at every pediatric epilepsy center [59].

In the 1920s and 1930s, all preliminary reports that verified the effectiveness of the ketogenic diet were retrospective reports [37,44,61,62,63,64,65,66,67,68]. Unfortunately, some of these included a small number of patients and few clinical details and it was often not clear what should be regarded as a “good” or “partial” response to the ketogenic diet. Despite these limitations, the literature supports that seizure control can be improved by adopting the ketogenic diet in some children. 

In later years, specifically in 1999, Sirven et al. carried out a modern, prospective study to assess the ketogenic diet efficacy and safety in treating adult patients with intractable, symptomatic partial, or generalized epilepsy [69]. The patients of this study suffered from weekly to daily seizures and had failed an average of 5.4 AEDs. A greater than 50% reduction in seizure frequency was reported in 54.5% (6 of 11) of the patients at eight months of follow-up, while four patients discontinued the diet. Moreover, all seizure types responded to the diet, and most patients tolerated the diet, with significant improvement seen in some patients who had been initially considered as hopeless cases. Moreover, an update on the efficacy of the ketogenic diet in the modern era of antiepileptic drug therapy was reported in 1992 [70], in which the data of 58 consecutive patients who were placed on the ketogenic diet at Johns Hopkins Hospital during the 1980s was analyzed. That report documented that 67% of the patients on the ketogenic diet showed an improvement in seizure control and that 75% of these patients followed the diet for at least 18 months, which established that the diet is effective and palatable based on the patient’s desire to continue with it. On the other hand, the earliest multicenter study on the effectiveness of the classical ketogenic diet was finalized in 1998 [71,72], in which 51 children were prospectively enlisted through epilepsy foundations with regular assessment of their seizure rate at 3, 6, and 12 months after following the ketogenic diet. Results from the study showed that 10% of the patients were seizure-free at one year, while a higher than 50% decrease in seizure rate was attained in 40% of the patients. Thus, this study demonstrated the efficacy of the ketogenic diet in different epilepsy centers and with different support staff.

The ketogenic diet has become available at almost all major children’s hospitals within the last 20 years, even though the KD was used for the first time to treat children with epilepsy a century ago. Ultimately, all the academic and proper studies on the ketogenic diet efficacy in epilepsy can be employed as a model for future clinical studies to investigate the diet efficacy in other diseases. For examples, plenty of accepted feedback observed an improvement in the health condition and efficacy of KD in patients with metabolic defects, many types of malignancy, trauma, ischemia, and neurodegenerative disorders such as Alzheimer’s and Parkinson’s disease [73].

For cancer management, KD has been investigated as an adjuvant to cancer therapy in animal models and human case reports. For example, in early 1987, Tisdale et al. observed a reduction in tumor weight and improved cachexia in mice with colon adenocarcinoma xenografts consuming a ketogenic diet [74]. Other studies have shown that KDs reduce tumor growth and improve survival in animal models with malignant glioma [24,75,76], colon cancer [77], gastric cancer [78], and prostate cancer [79,80,81]. In addition, some of the clinical results include a case report of two female pediatric patients with advanced-stage malignant astrocytoma who showed a 21.8% reduction in tumor SUV when these patients followed a ketogenic diet [26].

## 3. Cancer Metabolism and Warburg Effect

Cancer metabolism refers to the alterations in cellular metabolism pathways that are evident in cancer cell in comparison with most normal tissue cells and is one of the fundamental hallmarks of cancer [82].

This characteristic and profound metabolic alteration is mainly driven by oncogenic signaling pathways and also by amplified or alternatively spliced metabolic enzymes, which allows cancer cell accommodation to metabolic demands needed to sustain cell growth, proliferation, and survival in an environment with fluctuating nutrient levels. Nevertheless, this alteration makes cancer cells dependent on a constant supply of nutrients and energy in addition to the studied deregulated glucose metabolism, which leads to the production of more amino acids and fatty acids, thus increasing fuel tumor cell growth [83,84].

A common characteristic of cancer cells is increased glucose uptake in order to support the production of intermediates needed for the synthesis of lipids, proteins, and nucleic acids. In addition, cancer cells via increased glutamine uptake and glutaminolysis are able to maintain a continuous supply of intermediates in the tricarboxylic acid (TCA) cycle that are diverted into biosynthetic reactions [85]. This increased biosynthetic activity also requires an accompanying increased production of NADPH, which serves as a reducing agent for anabolic reactions and to maintain cellular redox balance [86]. Furthermore, the epigenetic modifications that occur during the process of cellular transformation and cancer progression are derived from metabolites such as acetyl-CoA for acetylation, NAD for deacetylation, SAM for methylation, α-ketoglutarate for demethylation, and UDP-GlcNAc for glycosylation [87]. Moreover, recent advances in the carcinogenesis process have revealed that cancer is a complex disease and that simple investigation of genetic mutations of cancerous cell is not adequate to understand it, and that cancer cells are present in a complex tumor tissue, communicate with the surrounding microenvironment, and develop traits that promote their growth, survival, and metastasis [88].

### 3.1. Glutamines

Glutamine, which is the most abundant amino acid in plasma, serves as a nitrogen source for nucleotides, amino acids, and hexosamines and supplies both nitrogen and carbon by involvement in numerous reactions in the cell. Consequently, glutamine plays a critical role in the proliferation of cancer cell since nitrogen is an essential metabolite for nucleotide biosynthesis. Moreover, glutamine is a precursor for other non-essential amino acids (NEAAs) and fatty acids synthesis [89,90].

In addition, many cancer cells become dependent on glutamine, leading to what is called glutamine addiction, which is described as a high glutaminolytic flux rate, wherein the glutamine transporter, ASCT2 (SLC1A5), transfers glutamine into the cytoplasm. Furthermore, glutamine serves as a fuel for TCA cycle intermediates, which come out as mediators of malignant transformation in cancer cells; thus, glutamine is the principal source of NADH and FADH2 in cancer cells as a consequence of aerobic glycolysis and oxidative phosphorylation [91].

### 3.2. Serine and One Carbon

Serine, a non-essential amino acid, assists in various metabolic critical processes in the growth and survival of proliferating cells; for this purpose, serine is either synthesized from the metabolic intermediate produced from glycolysis 3-phosphoglyceric acid (3PG) or transported from the medium. Serine serves as an important one-carbon donor to the folate cycle, thus contributing to nucleotide synthesis, methylation reactions, and the generation of NADPH for antioxidant defense, which makes it the third most consumed metabolite by cancer cells after glucose and glutamine [92].

In view of the fact that many cancer cells are highly dependent on serine, this trait provides several novel therapeutic opportunities, either through the inhibition of de novo serine synthesis or by limiting the availability or uptake of exogenous serine. This role of serine has motivated the investigation of phosphoglycerate dehydrogenase enzyme (PHGDH) as a target for cancer therapy since it is involved in the synthesis of serine [93].

### 3.3. Leucine 

Leucine is a branched-chain essential amino acid (BCAA) in humans. It is obtained from different dietary sources that contain protein, such as meats, dairy products, soybeans, and other legumes [94,95]. Leucine has an essential role in protein synthesis and several metabolic functions. It is involved in the regulation of blood sugar levels, the growth and repair of muscle, and bone tissue as well as growth hormone production [96]. L-leucine is one of the ketogenic amino acids wherein the outcomes of their degradation are ketone bodies, specifically acetoacetate and its metabolite β-hydroxybutyrate [97]. Leucine activation of mTOR and S6K1 caused the prevention of insulin signaling and subsequently a reduction of glucose utilization in skeletal muscle [98]. It is also used as a fuel molecule after being converted by enzymes into isovaleryl-CoA and is used in the citric acid cycle (TCA) to produce ATP [99]. During cancer progression, cells need to utilize alternative energy molecules to compensate for the fast rhythm of growth [100]. Hence, a high plasma level of BCAAs, including leucine, was observed in pancreatic and melanoma patients [101,102]. Moreover, leucine-activated mTORC1 by supplying acetyl-CoA to the EP300 acetyltransferase, which moderates blocking acetylation of the mTORC1 regulator Raptor at K1097 [103]. Different types of cancer depend on m-TOR activity to keep their cellular growth and proliferation [104]. Dietary manipulation of amino acids was considered in cancer therapy. It was observed that leucine deprivation might reduce cell growth, stimulate apoptosis, and suppress lipogenic gene FASN expression in breast cancer [105]. L-type amino acid transporter (LAT1) is responsible for leucine influx into the cells. Thus, the inhibition of LAT1 means the suppression of mTOR signaling, and subsequently, the prevention of tumor growth [106,107]. However, several studies have shown the benefits of using a leucine-rich diet in malnourished and cachectic cancer patients [108,109,110].

### 3.4. Warburg Effect

Cancer cell metabolism is transformed for the purpose of promoting cellular growth, proliferation and long-term maintenance with increased glucose uptake and the fermentation of glucose to lactate being common features of such changes in metabolism, known as the “Warburg effect” [85].

“Otto Warburg “showed that cultured tumor tissue has high rates of both glucose uptake and lactate secretion, even in the presence of oxygen and functioning mitochondria. Additionally, Otto Warburg showed that tumors were taking up large amounts of glucose compared with normal surrounding tissue and glucose was fermented to produce lactate, which was called aerobic glycolysis. Aerobic glycolysis is the conversion of pyruvate to ATP and lactate, which is catalyzed by lactate dehydrogenase. Evidence also suggests that the production of lactate via the Warburg effect grants cancer cells a selective advantage over normal cells in their microenvironment [111,112]. Furthermore, lactate has been shown to be a key driver of angiogenesis and has a critical role in the development and growth of cancer cells; nevertheless, the causal relationship of the Warburg effect with cancer progression is still unclear [113].

## 4. Ketogenic Diet as Cancer Therapy: Mechanisms of Action

The ketogenic diet (KD) is defined as a high-fat, low-protein, and low-carbohydrate diet and has been used in the treatment of several diseases. Moreover, KD can be considered inexpensive, safer, and easier to be carried out when compared to traditional anticancer therapy. Currently, the ketogenic diet (KD) offers an encouraging chance to be used either as a therapeutic diet or as an adjuvant cancer therapy in animal models and humans. The following section will discuss dietary adjustments (i.e., ketogenic diet) which is expected to enhance chemotherapy effects on tumor cells, protect healthy cells, lower inflammation, and regulate gene expressions of different proteins and factors including (matrix metalloproteinases (MMPs), histone deacetylases (HDACs), AMP-activated protein kinase (AMPK), pyruvate kinase (PK), and P53 [20,21,114,115].

### 4.1. The Effect of a Ketogenic Diet in Warburg Effect

**Figure 1:**
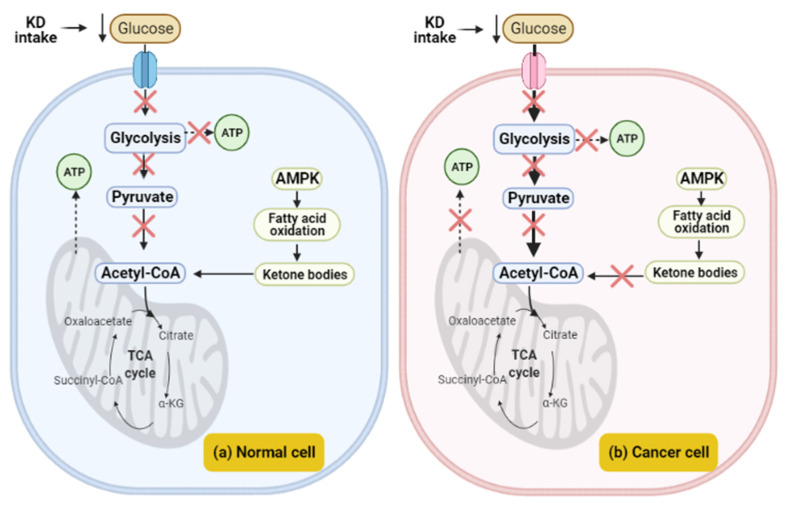
Scheme of cell behavior during ketogenic diet; (**a**) normal cell feeding with KD, lower level of glucose prevents glycolysis pathway, increases ketone body level as a result of fatty acid oxidation, thus enhancing level of acetyl-CoA in mitochondria to compensate for the ATP needing (**b**) cancer cell feeding with KD, and abolishes glycolysis. Additionally, the mitochondria are dysfunctional so the cell cannot produce ATP; thus, KD prevents cancer cell proliferation.

Cancer cells are characterized by metabolic disorders that lead to a high uptake of glucose. As discussed in Section 3, this metabolic alteration in cancer cells can be explained by a phenomenon called the Warburg effect, which is described as an increase in aerobic glycolysis to produce ATP. Moreover, in the absence of oxygen, the non-oxidation pathway plays a key role in ATP production in cancer cells by production of lactate from the fermentation of glucose. In addition, cancer cells display a high level of ROS production as a result of the electron transport chain being unworkable. On the basis of glucose being an essential component for tumors and completely dependent on it for survival and proliferation, the ketogenic diet is a dietary therapeutic strategy that decreases body glucose levels and conversely increases ketone body levels, which cannot be utilized by cancer cells as substrates for ATP production. Interestingly, KD causes the starvation of tumor cells and hence displays a great anti-cancer activity. On the contrary, for normal cells after prolonged exposure to KD intake and glucose limitation, fatty acid oxidation plays a key role in ketone body synthesis (including acetoacetate, beta-hydroxybutyrate, and acetone), which is then converted to Acetyl-CoA and enters the TCA cycle for ATP production; thus, ketone bodies help to maintain the normal cell in a general perfect health state (Figure 1) [116,117,118,119,120,121].

### 4.2. Ketogenic Diet and Inflammation

Inflammation is a term used to describe a series of responses of vascularized body tissues to injury as well as to chronic diseases. It involves many soluble factors including cytokine, chemokines, and other transcript factors and it has a real connection with cancer. While inflammation conditions are present before a malignant change occurs in some types of cancer, conversely, in other types of inflammation, it acts as a tumor promoter to enhance tumorigenesis, proliferation, metastasis, and also diminish the efficacy of treatment. The use of KD showed a reduced expression of pro-inflammatory cytokines (TNF-α, IL-1β, and IFN-γ). These anti-inflammatory effects of KD may enhance the response to the treatment and prevention of cancer [122,123].

#### 4.2.1. Tumor Necrosis Factor Alpha (TNF-α)

Tumor necrosis factor alpha is a pro-inflammatory cytokine that plays a central role in the pathogenesis of rheumatoid arthritis. Moreover, TNF-α is chronically produced in various types of cancer [124,125]. In cancer, TNF-α acts directly to enhance tumor growth and proliferation, stimulating angiogenesis, invasion, metastasis, and DNA damage [123,126]. Meanwhile, TNF-α induces the release of chemokine (such as IL-8) and activates NF-kB transcription factors, which play an essential role in cancer progression [123,127]. Several studies in glioblastoma (GBM) have shown that applying a drug–diet combination of KD and 6-diazo-5-oxo-1-norleucine (DON), a glutamine antagonist, has a significant effect on decreasing tumor growth, increasing the survival rate and reducing inflammation [91,128,129]. Moreover, Mukherjee et al. reported that the combined therapy of KD/DON in a syngeneic GBM mouse model, revealed a reduction in TNF-α expression, cell proliferation, and enhanced DON activity [130]. TNF-α also exhibits a multi-function in breast cancer such as tumor markers, promotion, progression, and metastasis [131,132,133]. Khodabakhshi et al., in a randomized control trial on breast cancer patients, showed a significant diminish of TNF-α level after 12 weeks of KD intake that is explained by the suppression of MMP-9 expression [134] and activation of PPARγ [135,136].

#### 4.2.2. Cyclooxygenase (COX)

Cyclooxygenase (COX) is a key enzyme involved in the production of prostaglandins and other eicosanoids from arachidonic acid [123], with two COX isoforms—COX-1 and COX-2—identified [126]. Overexpression of COX-2 has been reported in several human malignancies, including colon cancer [137,138], breast cancer [139,140], and lung cancer [141]. The COX-2 isoform critically influences all stages of tumor development from tumor initiation to tumor progression; hence, selective COX-2 inhibitors may potentially be effective in either preventing or treating cancer. Results from a mouse glioma model in- vitro study have shown that the KD markedly decreased COX-2 expression, while other research studies which combined the KD to radiation therapy showed a potentially reduced expression of COX-2 and other inflammatory factors [22,24,142]. In order to confirm the actual efficacy of KD in the attenuation of the expression of COX-2, more research and future studies are needed.

### 4.3. Ketogenic Diet and Matrix Metalloproteinases (MMPs)

Matrix metalloproteinases (MMPs) are a group of zinc-dependent endopeptidases enzymes which are the key enzymes responsible for the breakdown of the extracellular matrix (ECM) [143], which is responsible for holding cells together and maintaining the three-dimensional structure of the body. Moreover, several MMPs, besides their role in the degradation of extracellular matrix proteins, also play a role in cancer progression, migration, invasion, metastasis, and angiogenesis [144]. Studies have shown an overexpression of MMP-1, 2, 3, 7, 9, and 13 in human colorectal cancer [144], glioblastoma [142], and gastric cancer [145]. Additionally, MMP-9 comprises the gelatinase sub-family of MMPs and is primarily produced by inflammatory cells and from the stromal cells surrounding a tumor or by the cancer cells themselves [146]. The use of ketogenic diet, in combination with other conventional cancer therapy, is expected to provide a noticeable reduction in the expression of MMP-9 in different types of cancer [142,147].

### 4.4. Ketogenic Diet and Histone Deacetylases (HDAC)

HDACs is a critical family of proteins involved in the transcriptional regulation of a large number of genes and in the functional regulation of multiple proteins, and also catalyze the deacetylation of specific lysine residues in DNA-bound core histone protein [148]. Moreover, these histone deacetylases play a central role in the regulation of several cellular properties which are very closely linked with the development and progression of cancer [149]. Consequently, HDAC inhibitors have been shown to induce specific changes in gene expression and to influence a variety of other processes, including growth arrest, differentiation, cytotoxicity, and the induction of apoptosis [150]. Although numerous correlative studies have demonstrated the relationship between the KD and ketone bodies and suppression of the expression of HDACs in human tumors, more studies are required in this field [21,147,151].

### 4.5. Ketogenic Diet and Pyruvate Kinase (PK)

Pyruvate kinase (PK) is a terminal glycolytic enzyme that catalyzes the production of ATP and pyruvate by transferring phosphoenolpyruvate (PED) to adenosine diphosphate (ADP) [147]. Among the four encoded PK isoforms, pyruvate kinase M2 (PKM2) is the one expressed in cancer cells and has a major role in cancer metabolism [152]. The PKM2 isoform is predominately found in liver cancer, colon cancer [153], lung cancer [153], breast cancer [154], and renal cell carcinoma [155]. Zhang et al.’s study on colon cancer showed that KD attenuated the expression of PKM2, thus reducing glucose uptake and lactate production in the tumor cell, which destroys the Warburg effect, which is considered a survival method for cancer cells [147]. In addition, the PKM2 isoforms play a key role in tumorigenesis, cell proliferation, and hypoxia-inducible factor-1alpha (HIF-1alpha) function [152]. Beta-hydroxybutyrate (BHB), which is a stable component of ketone bodies, is produced in the brain via both the oxidation of fatty acids and catabolism of amino acids in astrocyte cell, also by crossing the blood–brain barrier (BBB) and entering the brain [130]. Furthermore, ketone bodies are currently being investigated as an adjunct in anti-glioma therapy. Chen JI et al. showed that BHB inhibited glycolysis by attenuating the expression of PKM2, thus reducing the amount of ATP production and promotion of apoptosis GMB [156].

### 4.6. Ketogenic Diet and P53

P53 is a nuclear transcription factor localized within the cell nucleus that acts as one of the tumor suppressor factors with a fundamental role in the control of cell proliferation, apoptosis, and genetic stability [157,158]. In normal cells in healthy individuals, P53 is expressed at an extremely low level and is degraded by protein ligase MDM2 [158]. Nevertheless, mutations in the transcription factor p53 are a fact in most cancers, thus resulting in the accumulation of altered p53 proteins with a prolonged half-life, which leads to therapy resistance [73,159]. Observations in several studies have indicated the importance of KD in the downregulation of p53 mutants through de-acetylation and the induction of cell death. This reduction of mutant p53 expression increases the lifespan. Moreover, a diet with a low glucose level leads to p53 mutant deacetylation and degradation; as a result, the KD either blocks p53 mutant activity or silences the expression during malignant initiation and progression [21,160,161].

### 4.7. Ketogenic Diet and AMP-Activated Protein Kinase (AMPK)

**Figure 2:**
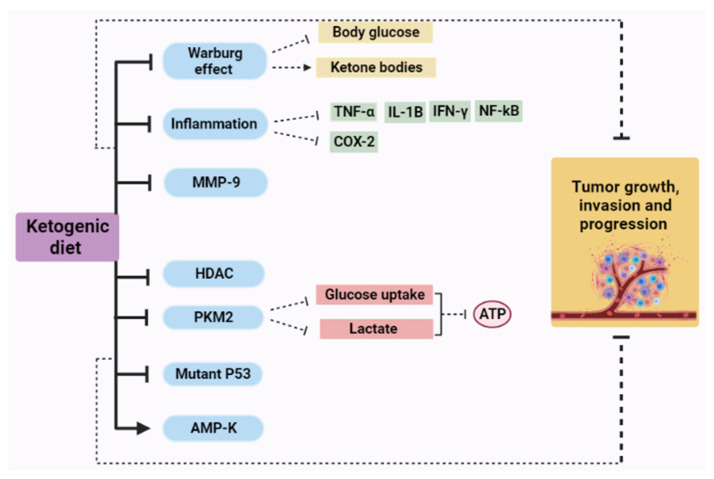
Ketogenic diet mechanism of action as a cancer therapy, (→), activation; (┬), inhibition; MMP-9, matrix metalloproteinases-9; TNF-α, tumor necrosis factor alpha; HDAC, histone deacetylases; PKM2, pyruvate kinase M2; AMP-K, AMP-activated protein kinase; IL-1B, interleukin 1 beta; IF-γ, interferon gamma; NF-kB, nuclear factor kappa B.

AMP-activated protein kinase (AMPK) is a serine/threonine-protein kinase, which is located in several types of cells [162]. Targeting AMPK offers a potential method to treat various types of cancer such as colorectal, lung, and liver cancer [163]. The activation of AMPK is associated with tumor suppressor genes including p53 and LKB1, suppression of cell proliferation, overcoming inflammation, cell growth inhibition, and cell cycle arrest; thus, AMPK plays a fundamental role in cancer prevention [163,164]. AMPK is activated in response to energy depletion under several conditions such as glucose deprivation and hypoxia. Moreover, the use of metformin, curcumin, quercetin, and some non-steroidal anti-inflammatory drugs can also activate AMPK [164,165,166]. Moreover, the exchange of glucose by ketone bodies in tumor cells as a result of keto diet intake was found to correlate with the amplified activation of AMPK [21,151,160]. Figure 2 summarize the mechanisms of action of KD in cancer therapy.

## 5. Ketogenic Diet as a Prevention of Cancer

Cancer cells undergo various metabolic modifications to satisfy their need for energy, glucose, protein, and signaling to proliferate. Otto Warburg described that cancer cells require more glucose than normal cells to generate ATP [167]. Cancer activates several pathways to survive. Moreover, carcinogenesis is mediated by different agents including the high level of blood glucose, insulin, inflammatory, and pro-inflammatory factors [168,169].

Multiple lines of research suggest the use of ketogenic diets (KD) or, more broadly, high fat, low carbohydrate, and sufficient protein diets as cancer treatment or prevention methods, either alone or in combination with medicines [168,170]. Several studies have looked into the connection between diet and reducing the risk of chronic diseases, such as cancer and age-related diseases, as well as extending the lifespan [171]. Dietary changes target multiple pathways, including the insulin pathway, PI3K, AKT, mTOR, ketone bodies, adiponectin and leptin protein distribution, and IGF-1 [167,169,172,173,174]. Preclinical and clinical studies have demonstrated the anti-aging and anticancer effects of KD [175,176,177,178,179]. Physical exercise, in addition to diet management, has been shown to reduce cancer risk in most cancer types.

PI3K/Akt dysregulation is directly associated with neoplasmic development, as well as increased resistance to cancer therapy, although PI3k promoted the downstream of both insulin receptor and IGF-1R [180]. The mechanistic (or mammalian) target of rapamycin (mTOR) is a serine-threonine protein kinase; mTOR signaling is regulated under a wide range of factors and circumstances [180]. It is stimulated by growth factors, mitogens, PI3K, activated AMP kinase, and hormones, including insulin [180]. Under low nutritional conditions, AMP-activated protein kinase (AMPK), phosphatidylinositide 3-kinase (PI3K), and mTOR are all acutely affected [171]. KD stimulates the AMPK signaling pathway, the tumor suppressor activity, which leads to mTOR signaling inhibition [171,180].

One of the main concerns regarding a high-fat diet is the potential to induce inflammation due to the high amount of fats, especially saturated fats [173]. While various fat types can lead to pro- or anti-inflammatory responses, saturated fat mimics the actions of lipopolysaccharide (LPS), which causes inflammation when it binds with its receptor on the surface of macrophages/monocytes and other innate immune cells [173,181]. In contrast, polyunsaturated fats such as the omega-3 fatty acids, EPA and DHA, have been found to have anti-inflammatory properties [173,182]. Chronic inflammation is described as an increase in the release of inflammatory cytokines into the local and systemic circulation. Recently, inflammation has been considered a characteristic of cancer [183,184]. In many tissues and tumor types, a low carbohydrate, high-fat diet (with a concentration on unsaturated fats) reduces the amount of tumor-infiltrating macrophages, the levels of circulating and tissue cytokines, the NF-B signaling, and COX-2 expression [171]. Thus, the high inti-inflammatory activity of KD implemented a cancer prevention effect [171].

The metabolic outcome from consuming a ketogenic diet is the formation of ketone bodies, including hydroxybutyrate (β-HB), acetoacetate, and acetone [173,185]. A high concentration of β-hydroxybutyrate triggers an uncoupling protein-2 (UCP-2) in mitochondria [169], which protects cells from oxidative stress. On the other hand, its absence may cause an abundance of reactive oxygen species, the release of pro-inflammatory cytokines, and persistent activation of nuclear factor kappaB (NF-κB) [169,185]. Thus, ketone bodies play a critical role in decreasing oxidative stress and extending the patient life cycle [173,185].

Adiponectin, leptin are peptide hormones produced from visceral white adipose tissue. Adiponectin has a negative correlation with leptin and other adipokines. Lower levels of adiponectin have been linked to type 2 diabetes, insulin resistance, metabolic syndrome, hypertension, cardiovascular diseases, and cancer. Several studies have demonstrated the protective role of the keto diet in decreasing the risk of cancer, reducing oncogenesis hormones, and extending the lifespan. Additionally, increased adiponectin, which activates several pathways such as AMPK, MAPK, and PI3K/Akt also reduces pro-inflammatory cytokine expression [171,186].

Finally, a direct connection was established between a high-calorie diet and the risk of cancer, as well as a way to proceed for cancer prevention by lifestyle modification such as physical activity and exercise, and healthy diets rich in fruit, vegetables, and whole grains, and low in red meat and saturated fats [185]. KD may not prevent the occurrence of a tumor, but it delays tumorigenesis and improves the survival rate [170,173]. Moreover, KD shows a synergistic effect on cancer treatment when combined with chemotherapy or other cancer therapies [170,180].

## 6. Ketogenic Diet as an Epigenetic Modifier in Cancer 

Epigenetic modifications represent an essential step of gene transcription regulation. It has been observed that DNA methylation, miRNAs, and histone modifications occurred during the early stages of cancer progression and metastasis [187,188]. Recent studies have suggested that ketone bodies can organize cellular functions through innovative epigenetic modulation; β-hydroxybutyrylation, which integrates the DNA methylation; and histone covalent post-translational modifications (PTMs) [189,190]. Interestingly, using a ketogenic diet enhanced adenosine production, which resulted in the blocking of DNA methylation [191,192]. Moreover, ketone bodies (β-hydroxybutyrate and acetoacetate) have an impact on epigenetic factors. They restrain the histone deacetylase 1 (HDAC1) activity, and promote PTMs of proteins by butyrylation, affecting DNA methylation and acetylating histone and non-histone proteins [187,192,193,194,195].

MicroRNAs (miRNAs) are endogenous small non-coding RNA sequences approximately 22 nucleotides in length [196,197,198] that regulate gene expression by binding with the target mRNA to regulate protein synthesis or degradation of the mRNA [199,200,201,202]. MicroRNA plays a part in a wide array of biological activities that involves cell differentiation, proliferation, death, metabolism, and balance of energy [196,203,204]. Various types of miRNAs have been linked to chronic disorders such as obesity, diabetes, and cancer [196,202,203,204], suggesting that they may operate as oncogenes or tumor suppressor genes [205]. MicroRNAs have been linked to cancer at all phases, including initiation, progression, apoptosis, angiogenesis, proliferation, and differentiation [197,201,206,207].

The miR-21 gene can be detected in the blood, bone marrow, liver, lung, kidney, gut, colon, and thyroid [208]. Many biological processes, including inflammation, fibrosis, and cancer, are controlled by miR-21 [209]. MicroRNA-21 (miR-21) is an oncogenic miRNA that is typically elevated in hepatocellular carcinoma (HCC) [210,211,212,213]. It promotes the release of inflammatory substances such as interleukin 6 (IL-6) [214], signal transducer, and activator of transcription 3 (STAT3)-dependent mechanism [215]. It also modulates growth factor (TGF-) via the SMADs signaling cascade [216]. Furthermore, MiR-21 contributes to cancer progression by targeting tumor suppressor mRNAs such as tropomyosin 1 [217], programmed cell death 4 (PDCD4) [218], phosphatase, and tensin homolog (PTEN) [219].

MiR-21 levels are also higher in the serum and plasma of multiple myeloma (MM) patients, considered to be used as a biomarker for the MM [220,221,222,223]. MiR-21 controls the expression of genes involved in MM proliferation, the G1/S transition, and invasion [224,225]. The KD significantly alters the expression of several microRNAs on tumor tissue from animals fed the KD versus those fed a standard rodent diet [199,226].

In breast cancer patients, the expression of various miRNAs discriminated tumors from normal tissue. MiR-10b, miR-125b, let-7, and miR-145 were significantly downregulated in malignant tissue, but miR-21 and miR-155 were overexpressed [197,227]. Another study found that overexpression of miR-335, miR-126, and miR-206 reduced metastasis from the breast to the lung or bone in mice [228,229]. INF- promotes the proliferation and spread of breast cancer cells through promoting the production of miRNA-23b and miRNA-27b, which is widely recognized as a hallmark of cancer [135]. According to many studies, the keto diet appears to have anti-inflammatory characteristics by contributing to the reduction of -TNF- expression through PPAR activation [135,230].

MiRNA changed several tumor-suppressive and oncogenic pathways connected to colorectal cancer (CRC), including Let-7, MiR-21, and MiR-145 [198]. In colorectal cancer cells, Let-7 miRNA operates as a tumor suppressor miRNA, influencing the expression of the Ras and c-myc genes, which are both critical in colon cancer development and progression [231,232,233]. In addition, the Let-7 miRNA regulates p53 [234,235,236]. Oncogenic miRNAs, such as MiR-21, are overexpressed in colon tumor tissues. MiR-21 is designed to restrict the expression of the phosphatase and tensin homolog (PTEN) gene; however, a later study has revealed that it also suppresses other tumor suppressors such as programmed cell death 1 [201,237,238,239]. The role of miR-145 in colorectal cancer appears to be debatable. While it was previously thought to be a tumor suppressor miRNA in colorectal cancer due to its ability to target both the insulin receptor substrate-1 and the insulin-like growth factor receptor 1 (INF-1), more recent studies have shown that upregulation of miR-145 can improve the ability of colorectal cancer cells to migrate and invade [201,240,241]. Environmental and lifestyle factors are prevalent causes of colorectal cancer [201]. One such element was that diets have been demonstrated to alter miRNA expression in patients with colorectal cancer [201].

MiRNAs have been demonstrated to affect many elements of the development of cancer and metastasis which can be used as biomarkers and therapeutic targets. Diverse dietary products, including nutrients (vitamins, minerals, fatty acids, etc.) and bioactive foods (curcumin, resveratrol, catechins, etc.), protect against cancer through modulating the expression of miRNA [197,201,222]. KD was also applied in animal and human models as an adjuvant cancer treatment. The impact of KD on reducing the development of the tumor and improving survival of malignant glioma models in animals has been demonstrated in preclinical trials by the modulation of miRs; this is also the case for prostate cancer, colon cancer, and gastrointestinal carcinoma [24,75,199,226,242]. KD increases the expression of several miRNAs, many of which have been shown to have tumor suppressor properties in glioma [202,243]. More in-depth mechanistic investigations are needed to determine the potential function of miRNA and the keto diet in the development of cancer.

## 7. Ketogenic Diet in Experimental and Clinical Anticancer Studies

The ketogenic diet (KD) offers an encouraging chance in the treatment of cancer by targeting metabolic alterations in tumor cells. This is based on recent research findings that showed potential effects of the KD in cancer including a growth-limiting effect on tumors, the protection of healthy cells against chemotherapy or radiation damage, promoting chemotherapeutic toxicity toward cancer cells, and a decrease in inflammation. Moreover, the KD, in comparison with anticancer drugs and standard treatments is inexpensive, well-tolerated and adequately easy to apply since many good recipes are available [244]. In addition, KD caused an increase in intra-tumor oxidative stress, and a KD-induced apoptosis against tumor cells was reported by some studies which lead to down regulation in the protein expression of matrix metalloproteinase-9 in some tumor-bearing mice [245].

### 7.1. Preclinical Studies

Dietary interference with the use of KD as an effective anticancer therapy has been suggested by an increasing number of preclinical studies in spite of reported pro-tumor effects or severe side effects in certain cancer models in some studies [246]. In most preclinical studies, KD slowed tumor growth, delayed the initiation of tumors, prolonged the survival rate, and reversed the mechanism of cancer-induced cachexia (extreme weight loss and muscle wasting) [247,248]. Many studies also showed that KD is able to increase the tumor response to classic chemotherapies or radiotherapies [249]. Moreover, a study on different mouse cancer models, including breast cancer, endometrial, bladder, pancreatic and acute myeloid leukemia, have shown that the KD enhanced the efficacy of targeted therapy—in particular, phosphatidylinositol-3 kinase (PI3K) inhibitors [27].

The PI3K pathway is one of the most frequently activated pathogenic signaling cascades in human malignancies. Moreover, the PI3K is genetically mutated or overexpressed in a wide variety of cancers, and results in drug resistance, which indicates that the KD keeping blood sugar levels low would improve the ability of PI3K inhibitors to treat cancer [27]. Consequently, the importance of enhancing the efficacy of KD by optimization of its composition was tackled by many studies either by increasing the percentage of fat or supplementing with omega-3 fatty acids, ketone esters, or MCTs [250,251], as shown in Table 1, which represents data from numerous preclinical studies.

#### Ketogenic Diet as an Experimental Glioma Therapy 

Maurer et al. carried out a study to explore if energy metabolism in tumor cells can be impaired by a ketogenic diet. In vitro, he studied the effects of the 3-hydroxybutyrate, principal ketone body, in both rat hippocampal neurons and five glioma cell lines. In in vivo studies, an orthotopic xenograft glioma mouse model was used to examine a ketogenic diet (compositions summarized in Table 2). The study showed that in vitro, glioma cells were not able of metabolizing ketone bodies in order to compensate for glucose restriction, suggesting a potential disadvantage of tumor cells compared to normal cells under a carbohydrate-restricted ketogenic diet. However, in the xenograft model used, an unrestricted ketogenic diet was not effective as a monotherapy; thus, further studies and investigations are necessary to identify co-treatment modalities, e.g., glycolysis inhibitors or antiangiogenic agents that efficiently target non-oxidative pathways [75].

### 7.2. Clinical Studies

Although the combined use of radiation, chemotherapy, and surgery is a good strategy of care in different cancers [258], no effective standard therapy is available for highly aggressive cancer types with poor prognosis—for example, triple negative breast cancer [263]. Therefore, there is an urgent need to develop new approaches or strategies that enhance therapeutic efficacy in these types of cancer.

Most of the clinical studies involving the KD have been focused on safety and tolerability; however, consistent findings include the induction of ketosis, a moderate reduction of blood glucose levels, feasibility and tolerability of the KD, as well as improvement in quality of life [259]; several individual observations that support the antitumor effects of KDs have been reported in humans (Table 3). KD decreases glucose levels, eliminating the benefits for cancer cells by glycolysis. These dietary strategies enhance ketones and other metabolites that normally interact with the mitochondrial ATP generation process and target cancer cells [119]. KDs that target the Warburg effect, without toxicity to normal cells, could starve cancer cells by decreasing fasting, postprandial blood glucose concentrations, reducing cancer cachexia, muscle waste, and fatigue. The Warburg effect can reduce the concentration of insulin and other growth-stimulating hormones and factors, improve immune modulation, and reduce the side effects of chemotherapy and radiation [160,264].

Based on the results from different studies mentioned above, the direct effects of KD on tumor development and growth can be demonstrated, and the KD also has the potential to improve the quality of life (QoL) of patients and their overall health statuses, besides the feasibility and safety of using a KD in cancer patients [277,278]. In addition, some studies reported a normalization or an overall improvement of lipid profiles, including a reduction of total cholesterol, LDL, and HDL cholesterol, in cancer patients on a KD [279,280]. Furthermore, the KD led to a significant reduction in insulin levels, and an inverse association between BHB and insulin-like growth factor 1 (IGF-1) concentrations was also reported [19]. Ketogenic diet would not be expected to be used as stand-alone treatment for cancer. However, this diet might act synergistically with other treatments, such as phosphoinositide 3-kinase inhibitors, chemotherapy, radiotherapy, and support prevention [264].

On the other hand, some studies were not able to reach any definitive conclusions regarding the efficacy of the KD in cancer patients, which may be explained either by poor compliance of cancer patients to the diet regime or lack of power of the study. However, in most cases, this poor compliance was attributed to either poor tolerability of the KD-associated adverse effects, including nausea, fatigue, or constipation, or to patients being unable to adhere to the diet because of the tumor progression [268,276]. Studies have also shown that fasting and following a KD results in a reduction of warranty effects of adjuvant chemotherapy (due to a reduction of chemicals and drug toxicity) and a better QoL in comparison with patients that follow no specific diet. Unfortunately, despite the fact that various animal and laboratory studies indicate advantages from KD and fasting, few data are usable today on humans [281].

## 8. Conclusions

Currently, the ketogenic diet term is gaining attention in the medical field, especially after the clarification of how cancer cells utilize nutrients to survive and proliferate. KD exhibits anti-cancer effects which are still partially elucidated, but has limited toxicity, low cost, and is easy to apply. KD may implement its anticancer activity via targeting tumor metabolism, the inflammatory process, gene transcription, and the tumor microenvironment. The published data of the preclinical and clinical trials support the use of a ketogenic diet as a preventive and adjuvant cancer therapy. KD intervention as an adjuvant therapy alongside conventional chemotherapy and radiation treatment can be adopted under certain protocols. However, compliance is a challenging problem for applying KD as a standard protocol in cancer therapy. More studies are still needed to fully evaluate the efficiency of KD against various cancer types and to test more combination therapies using KD.

## Figures and Tables

**Table 1:** Some of the preclinical studies for the effect of ketogenic diet on cancer (tumor) cells.

Cancer Type	Cell Line	Animal Model	KD Ratio	Study Group	Mechanism and Results of the Studies Compared with SD	Ref.
Glioblastoma	T98G, U87MG, NIH3T3, A172, LNT-229, U251MG	athymic nude mice	3:1	SD, KD	KD resulted in a significant increase in BHB (KB), but it had no effect on glioma cell lines, TP, BG levels, or survival → no effect.	[75]
	U87MG	athymic nude mice	3:1	SD ± CT, KD ± CT	KD alone: increase in KBs but no effect on TP or survival; KD+CT: increase the activity of CT drug → increase survival.	[252]
	GL261-Luc2	albino C57BL/6 mice	4:1	SD ± RT, KD, KD ± RT	KD alone: increase in BHB (KB) and survival; KD+RT: enhance antitumor additive effect from RT alone → increase survival.	[22]
	GL261-Luc2	albino C57BL/6 mice	4:1	SD, KD	The expression of VEGF receptor, MMP-2 and vimentin were reduced in tumors from animals on KD, and significantly reduced in the peritumoral edema.	[142]
Medullo-blastoma	Spontaneous tumor development	Ptch1/-Trp53/mice	4:1	SD, KD	KD reduced the insulin level and increased the KB level in mice but there was no effect on the TP or survival.	[253]
	Medulloblastoma from Ptch1/-Trp53/mice	NOD SCID mice	6:1	SD, KD	KD reduced the insulin level and increased the KB level in mice but there was no effect on the TP or survival.	[253]
Prostate cancer	LAPC-4	athymic nude mice	2:1	SD ± MCT1 inhibitor, KD ± MCT1 inhibitor	MCT1 inhibitor did not affect TP and increased necrotic fraction; KD decreased TP compared to SD and decreased the necrotic fraction.	[254]
	Spontaneous tumor development	transgenic Hi-Myc mice	2:1	SD, KD	KD worked as a protumor (preventive).	[255]
Colon cancer	colon 26	CDF1 mice	3:1	SD, KD	KD increased KB and decreased TP and plasma IL-6 levels compared with tumor-bearing mice taking SD.	[245]
	colon 26	BALB/c mice	4:1	SD, KD	The KD group showed an increase in survival and better health status, no effect on TP → KD good as a potential preventive therapy.	[246]
Pancreatic cancer	S2-013	athymic nude mice	2:1	SD, KD	KD caused reduced TP and inhibition of muscle and body weight loss by decreasing BG, glycolytic flux in tumor cells and increasing KB, which diminished glutamine uptake, overall ATP content, and survival of the pancreatic cancer cell lines, while inducing apoptosis of it.	[247]
	PANC-1	nu/nu mice	3:1	SD, KD	KD decreased TP and increased the survival rate by reducing energy supplies to cells, which damage the tumor microenvironment → antitumor effect.	[248]
	MIA PaCa-2	athymic nude mice	4:1	SD ± RT, KD ± RT	KD increased radiation sensitivity in a pancreatic cancer compared with radiation alone.	[249]
Breast cancer	Spontaneous tumor development	transgenic FVB MMTV-PyMT mice	4:1	SD, KD	KD decreased TP by suppressing tumorigenesis; this may perhaps reflect the inherent tumor-suppressive efficacy of free LCFA or their respective CoA-thioesters by suppressing their esterification into lipids due to limiting insulin and glycerol-3-phosphate.	[250]
	4T1	BALB/c mice	6:1	SD ± metformin, CR-KD ± metformin	KD enhanced the cytotoxic effect of metformin on tumor growth by decreasing ATP production and inhibiting survival signaling pathways.	[256]
	ES-272	C57BL/6 mice	6:1	SD ± PI3K inhibitors, KD ± PI3K inhibitors	KD enhanced PI3K inhibitors to decrease TP in tumor cell → increased the antitumor effect.	[27]
Lung cancer	LLC1	C57BL/6 (Fgf21 WT and KO) mice	3:1, 8:1	low-fat diet (SD), regular protein KD, low protein KD	Regular protein KD had no effect on TP but low protein KD showed decreased TP, i.e., an antitumor effect by the extreme increase of fibroblast growth factor 21 levels because of protein starvation.	[251]
	TC-1_luc	BALB/c mice	4:1	SD, KD, KD + mABs	KD increased BHB that slowed TP and induced a T cell-dependent anticancer effect.	[185]
	NCI-H292, A549	nu/nu mice	4:1	different experiments with different IR doses, but overall: SD ± RT, KD ± RT, SD + RT/CT, KD + RT/CT	KD enhanced the antitumor effect of RT that decreased TP compared with RT alone by a mechanism that may involve increased oxidative stress.	[257]
Melanoma	A375, A2058 (BRAF V600E)	nu/nu mice	4:1, 6:1	SD, KD	KD decreased glucose level and increased AcA, leading to increased TP → protumor effect.	[258]
	RET melanoma	C57BL/6JolaHsd BALB/c mice	4:1	SD, KD, KD + mABs	KD increased BHB that slowed TP and induced a T cell-dependent anticancer effect and KD had synergistic antitumor effects when combined with a combination of immunostimulatory mAbs.	[185]
Kidney cancer	786-O	CD-1 nude mice	8:1	SD, LCT-KD, MCT-KDs	KD reduced TP, but mouse survival was dramatically reduced due to massive weight loss in the KD group.	[259]
	RENCA-luc	BALB/c mice	4:1	SD, KD, KD + mABs	KD delayed the progression of TP, preventing tumor outgrowth in some mice and smaller tumors were observed in others.	[185]
	Spontaneous tumor development	Eker (Tsc2) rats	8:1	SD, KD	KD promoted TP by recruiting ERK1/2 and mTOR, which are correlated with the accumulation of oleic acid and the overproduction of growth hormone.	[260]
Liver cancer	DEN-induced hepatocellularcarcinoma	C57BL/6N mice	4:1	SD, KD	KD had no effect on TP.	[261]
	DEN-induced hepatocellularcarcinoma	C57BL/6N mice	5:1	low-fat/low-sucrose diet, KD, western diets, fructose diet	KD and a low-fat/low-sucrose diet feeding decreased TP compared with a high-sucrose diet; this effect correlated with sugar intake and was independent of excess adiposity or insulin resistance.	[262]
Uterus cancer	HeLa	nu/nu mice	3:1	SD, KD	KD showed an increase in TP and decreased survival because HeLa tumors actively consumed KB as an energy source.	[248]
	Patient-derived xenograft	nude mice	6:1	SD ± PI3K inhibitors, KD ± PI3K inhibitors	KD had no effect on TP alone but an enhanced antitumor effect of KD+PI3Kinhibitors compared with CD + PI3K.	[27]
Acute myeloid leukemia	MLL-AF9 Ds-Red	C57BL/6 mice	6:1	SD ± PI3K inhibitors, KD ± PI3K inhibitors	KD alone decreased the survival → protumor effect, but enhanced survival in KD + PI3K inhibitors group compared with CD + PI3K inhibitor.	[27]
Bladder cancer	Patient-derived xenograft	nude mice	6:1	SD ± PI3K inhibitors, KD ± PI3K inhibitors	KD alone decreased TP, and with PI3K inhibitors had an additive antitumor effect because the efficacy of PI3K inhibition can be limited in the presence of insulin feedback and in KD reduced levels of phosphorylated insulin receptor, decreasing the levels of tumor proliferation, increasing apoptosis, and enhancing PI3K inhibitors activity	[27]

2-DG: 2-deoxyglucose, AcAc: acetoacetate, BHB: b-hydroxybutyrate, CD: control diet, CHO: carbohydrate, CR-CD: calorie-restricted control diet, CR-KD: calorie-restricted ketogenic diet, CT: chemotherapy, DEN: diethylnitrosamine, DON: 6-diazo-5-oxo-L-norleucine, HBOT: hyperbaric oxygen therapy, IR: ionizing radiation, KD: ketogenic diet, KE: ketone ester, KO: knock out, LCT: long-chain triglyceride, LFD: low-fat diet, MCT: medium-chain triglyceride, MCT1: monocarboxylate transporter 1, NCKD: non carbohydrate ketogenic diet, PI3K: phosphatidylinositol-3 kinase, RT: radiotherapy, TP: tumor progression, WT: wild-type.

**Table 2:** Composition of the standard and ketogenic diets [75].

Component	Control, Standard Diet	Ketogenic Diet
Fat	6.1	35.5
Carbohydrate	55.6	0.2
Protein	21.8	13.0
Fiber	3.8	14.8
Ashes	5.3	2.1
Energy [kJ/g]	15.8	15.4
Ketogenic ratio	0.08:1	2.7:1

Components of the diets used are listed in grams per 100 g of food. The fat in the standard diet derived from soybean oil; the fat source of the ketogenic diet consisted of a mixture of vegetable oils from flaxseed and hempseed with elevated levels of omega-3 fatty acid and medium-chain triglycerides. The ketogenic ratio was calculated according to the following formula: fats/(protein + carbohydrates).

**Table 3:** Some of the clinical studies for the effect of ketogenic diet on cancer (tumor) cells.

Cancer Type	Study Group Size (n)	Dietary Intervention (n)	Combined with Tumor Therapy (n)	Study Duration	Results of the Studies	Effect on QoL	Ref.
Glioblastoma	20	KD 60 g CHO/day	ST as RT, CT, or antiangiogenic treatment	6+ weeks	PFS was observed in patient with stable ketosis (8); one with complete response and five with partial response.	Three patients stopped KD because they felt that KD decreased their QoL but there were no serious side effects.	[252]
	53	KD 30–50 g CHO/day (5), CR-KD (1)	RT	3–12 months	No tumor recurrence was observed on CR and KD patients after 12 months from RT.	Not specified.	[265]
Glioblastoma and gliomatosis cerebri	9	KD 4:1 (5), CD (4)	ST	2–31 months	This study shows the accumulation of Acn and AcAc in the brain in patients with brain tumors on KD; KD may have potential as a treatment given the metabolic changes.	Not specified, but some of the patients stopped KD because they felt that KD decreased their QoL.	[266]
Glioma	172	modified KD 70% kcal fat, 20 g CHO/day (6)	ST	3 months	KD appears to be a good adjuvant therapy; no data on TP.	Self-reported good QoL, but two patients reporting constipation, which was resolved through dietary changes.	[267]
	8	MAD 20 g CHO/day (8)	ST	2–24 months	KD increased the control of seizure in brain tumor patients. Increase in the survival rate.	Not specified.	[268]
	13 6 (newly diagnosed) 7 (recurrent)	KD + MCT + Metformin 850	RT (60Gy) for recurrent RT (35Gy) for newly	6 weeks (recurrent) 2 weeks (newly diagnosed)	Increase in survival rate. Synergistic interaction between radiation therapy and KD. Metformin has in-vitro anti-cancer activity through AMPK activation and mTOR inhibition.	Five patients stopped KD + MCT. Metformin 850 mg three-times daily was poorly tolerated.	[269]
Invasive rectal cancer	359	KD ≥ 40% kcal fat and <100 g/day glycemic load (48)	RT (18/48)	not specified	KD significantly reduced risk of cancer-specific deaths compared with NSAIDs cancer-specific death, smoking, or other diseases.	Not specified.	[270]
Breast cancer	1	strict KD + high dose vitamin D3, not further specified (1)	No	3 weeks	KD + vitamin D caused changes in biological markers of breast cancer (negativization of HER2 expression and increased expression of PgR).	Not specified.	[271]
	29 (on KD) 30 (on SD)	KD, SD	RT	5–6 weeks	The increase of TP was less pronounced in the KD group compared to the SD group (KD enhanced the RT effect).	Improvements in emotional functioning, social functioning, sleep quality, and side effects.	[272]
Triple-negative breast cancer (TNBC)	1	KD	MSCT + HT + BHO	6 months	KD effective in treating advanced TNBC clinical, radiological, and pathological complete with good response.	Self-reported increase in QoL.	[273]
Lung and pancreatic cancer	9 7 (lung cancer) 2 (pancreatic cancer)	KD 4:1 90% of calories from fat, 8% from protein and 2% from carbohydrate with a 4:1 ratio of fat to combined protein and carbohydrate	ST	5–6 weeks	Four patients were unable to comply with the diet and withdrew, two completed the study and one was withdrawn due to a dose-limiting toxicity. Two pancreatic patients—one completed the study, and the other was withdrawn due to a dose-limiting toxicity.	Difficulty for adults to comply with a ketogenic diet while receiving concurrent RT and CT.	[249]
Non-small cell lung cancer	44	mild KD, avoidance of high CHO foods	MSCT + HT + HBOT	6 months	KD with CT and HBOT improved survival outcomes and increased treatment response rates by targeting several corresponding metabolic pathways and weaknesses of cancer cells.	Not specified.	[274]
Ovarian and endometrial cancer	73	KD 70% kcal fat, 30% kcal CHO + protein (37), CD (36)	ST	3 months	Increase in ketone bodies with increase in physical function. KD group without chemotherapy reported significant increase in energy at 12 weeks follow up, no data on TP.	KD does not diminish QoL; KD may even increase QoL.	[275,276]
Head and neck cancer	12	KD, not further specified	not specified	7 days	Increase in body weight. No data on TP.	Not specified.	[277]

BHB: b-hydroxybutyrate, CD: control diet, CHO: carbohydrate, CR: calorie restriction, CR-KD: calorie-restricted ketogenic diet, CT: chemotherapy, EAA: essential amino acids, GTPN: glucose-based total parenteral nutrition, HBOT: hyperbaric oxygen therapy, HER2: human epidermal growth factor receptor 2, HT: hyperthermia, KD: ketogenic diet, LCHF: low-carbohydrate high-fat diet, LTPN: lipid-based total parenteral nutrition, MAD: modified Atkins diet, MSCT: metabolically supported chemotherapy, OS: overall survival, PFS: progression free survival, PgR: progesterone receptor, POH: perillyl alcohol, PR: partial response, QoL: quality of life, RT: radio therapy, SD: stable disease, ST: standard therapy, TKTL1: transketolase-like-1, TP: tumor progression, TR: tumor regression.
